# Reactive Microneedle Patches with Antibacterial and Dead Bacteria‐Trapping Abilities for Skin Infection Treatment

**DOI:** 10.1002/advs.202309622

**Published:** 2024-04-06

**Authors:** Jingyang Shan, Xiangyi Wu, Junyi Che, Jingjing Gan, Yuanjin Zhao

**Affiliations:** ^1^ Department of Rheumatology and Immunology Nanjing Drum Tower Hospital School of Biological Science and Medical Engineering Southeast University Nanjing 210096 China; ^2^ Key Laboratory of Organic Electronics and Information Displays Jiangsu Key Laboratory for Biosensors Institute of Advanced Materials (IAM) Nanjing University of Posts and Telecommunications Nanjing 210023 China

**Keywords:** eliminate dead bacteria, microenvironment, microneedle, reactive metal boride, skin infection

## Abstract

Bacterial skin infections are highly prevalent and pose a significant public health threat. Current strategies are primarily focused on the inhibition of bacterial activation while disregarding the excessive inflammation induced by dead bacteria remaining in the body and the effect of the acidic microenvironment during therapy. In this study, a novel dual‐functional MgB_2_ microparticles integrated microneedle (MgB_2_ MN) patch is presented to kill bacteria and eliminate dead bacteria for skin infection management. The MgB_2_ microparticles not only can produce a local alkaline microenvironment to promote the proliferation and migration of fibroblasts and keratinocytes, but also achieve >5 log bacterial inactivation. Besides, the MgB_2_ microparticles effectively mitigate dead bacteria‐induced inflammation through interaction with lipopolysaccharide (LPS). With the incorporation of these MgB_2_ microparticles, the resultant MgB_2_ MN patches effectively kill bacteria and capture dead bacteria, thereby mitigating these bacteria‐induced inflammation. Therefore, the MgB_2_ MN patches show good therapeutic efficacy in managing animal bacterial skin infections, including abscesses and wounds. These results indicate that reactive metal borides‐integrated microneedle patches hold great promise for the treatment of clinical skin infections.

## Graphical abstract

Current strategies are mainly focused on the inhibition of bacterial activation for treating skin infections while ignoring the effect of an acidic microenvironment and dead bacteria remaining in the body. Here, a novel and interesting reactive microneedle patch with antibacterial and dead bacteria‐trapping abilities is designed to regulate the acidic microenvironment and remove dead bacteria for treating skin infections.

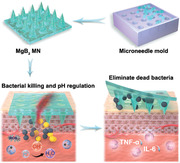

## 1 Introduction

Skin infections pose a growing threat to human health with their increasing prevalence.^[^ ^1^, ^2^, ^3^ ^]^ Once bacterial skin infections occur, the presence of bacteria can create an unfavorable environment for tissue repair and hinder the natural healing process.^[^ ^4^, ^5^, ^6^ ^]^ Traditional drug administration lacks tissue specificity, leading to reduced therapeutic effects and increased risks of systemic toxicity.^[^ ^3^, ^7^, ^8^ ^]^ Currently, there has been a growing interest in developing topical antimicrobial agents, such as antibacterials and nanomaterials.^[^ ^9^, ^10^, ^11^, ^12^, ^13^ ^]^ However, the therapeutic effects are still limited due to their primarily superficial action on the skin without effective penetration into deeper tissues. In addition, these materials have demonstrated bacteria‐killing properties, while ignoring the undesirable immune response triggered by dead bacteria.^[^ ^14^, ^15^, ^16^, ^17^ ^]^ Generally, these dead bacteria can induce systemic inflammation by releasing massive amounts of endotoxin, which is a major contributor to mortality associated with infectious diseases.^[^ ^18^, ^19^ ^]^ Thus, the development of effective therapeutic strategies that can enhance drug penetration, eliminate dead bacteria, and reduce local inflammation holds immense significance.

**Figure 1:**
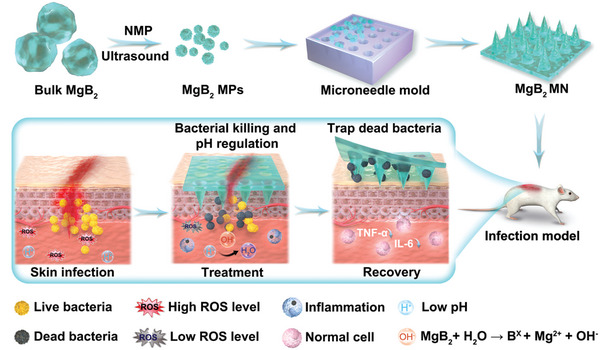
Schematic illustration of MgB_2_ microparticles (MPs) integrated microneedle (MgB_2_ MN) patches with multiple functions to kill bacteria, eliminate dead bacteria, and regulate pH microenvironment for treating bacterial skin infection.

In this study, we propose a reactive metal borides‐integrated microneedle patch with dual functions to eliminate dead bacteria and manage skin infections (**Figure** **1**). Reactive metal borides exhibit rich bonding characteristics that enable the release of metal ions, hydroxide, and the generation of boron hydride during hydrolysis.^[^ ^20^, ^21^, ^22^ ^]^ Through this process, the released hydroxides effectively regulate the configuration transition of boron atoms, forming stable borate esters by binding with key components such as lipopolysaccharide (LPS) or peptidoglycan (PGN) in dead bacteria.^[^ ^23^, ^24^, ^25^, ^26^ ^]^ Based on this principle, reactive metal borides demonstrate significant antibacterial capacity and show substantial potential in the treatment of bacterial infection‐related diseases.^[^ ^27^, ^28^, ^29^ ^]^ Nevertheless, the lack of a rational and effective delivery pathway limits their application in skin infection management. In contrast, microneedle (MN) has emerged as a promising approach for transdermal drug delivery, overcoming the skin barrier with minimal invasiveness and enhanced drug permeation.^[^ ^30^, ^31^, ^32^, ^33^, ^34^ ^]^ MN can accurately deliver antimicrobial drugs to deep skin tissues, concentrating them at the infection site, thereby effectively controlling the infection.^[^ ^35^, ^36^, ^37^ ^]^ Therefore, integrating reactive metal borides and MN patches to regulate the acidic microenvironment, kill bacteria, and simultaneously eliminate dead bacteria for skin infection treatment is urgently required.

Here, we introduced a magnesium boride (MgB_2_) microparticles integrated microneedle (MgB_2_ MN) for skin infection management. The MgB_2_ microparticles with defect‐rich active edges were prepared by a simple top‐down method. The resultant MgB_2_ microparticles could produce a local alkaline microenvironment by hydrolysis, which could promote the proliferation and migration of fibroblasts and keratinocytes.^[^ ^38^, ^39^ ^]^ Moreover, MgB_2_ microparticles exhibited remarkable bactericidal activity against *Escherichia coli* (*E. coli*), *Staphylococcus aureus* (*S. aureus*), and methicillin‐resistant *S. aureus* (MRSA) with a bacterial inactivation rate of 5 log. Moreover, the MgB_2_ microparticles could effectively mitigate dead bacteria‐induced inflammation through interaction with lipopolysaccharide (LPS). Based on these features, we have demonstrated that the MgB_2_ MN patch could effectively kill bacteria, trap dead *E. coli* and MRSA, and eliminate these dead bacteria from the solution. Through animal experiments, we have further revealed that the MgB_2_ MN patch could effectively treat MRSA‐infected subcutaneous abscesses and wounds, displaying excellent germicidal capacity and suppressing inflammation triggered by dead bacteria. Thus, we believe that the MgB_2_ MN patch holds promising prospects for the management of various skin infectious diseases and exhibits significant potential for clinical applications.

## 2 Results

### 2.1 Characterization of Defect‐Rich MgB_2_ MPs

**Figure 2:**
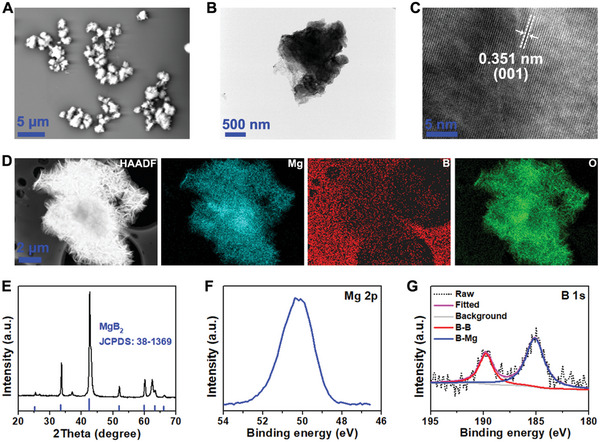
Characterization of defect‐rich MgB_2_ MPs. A) SEM image of MgB_2_ MPs. B) TEM image of MgB_2_ MPs. C) HRTEM image of MgB_2_ MPs. D) STEM and elemental mapping images of MgB_2_ MPs. E) XRD pattern of MgB_2_ MPs. F,G) XPS spectra of (F) Mg 2p and (G) B 1s orbitals for MgB_2_ MPs.

Defect‐rich MgB_2_ MPs were prepared by a top‐down method using bulk MgB_2_ materials (Figure S1, Supporting Information). Scanning electron microscopy (SEM) image revealed the rough surface of MgB_2_ MPs (**Figure** **2**A). The defect‐rich active edges of MPs were captured by transmission electron microscopy (TEM, Figure 2B). Furthermore, the high‐resolution TEM (HRTEM) image showed a clear crystal lattice spacing of 0.351 nm, which belonged to the (001) plane of MgB_2_ (Figure 2C). The high‐angle annular dark‐filed scanning TEM (HAADF‐STEM) image further provided a detailed view of the morphology of MgB_2_ MPs, highlighting the presence of defect‐rich active edges. Elemental mapping images confirmed the identical element distribution of Mg, B, and O in the as‐prepared MgB_2_ MPs (Figure 2D).

The X‐ray diffraction (XRD) pattern exhibited diffraction peaks corresponding to the planes of MgB_2_ (JCPDS: 38–1369) (Figure 2E). X‐ray photoelectron spectroscopy (XPS) was applied to characterize the chemical compositions and valence states of MgB_2_ MPs. The Mg 2p signal of the defect‐rich MgB_2_ MPs exclusively corresponded to Mg ions at 50.5 eV (Figure 2F). The characteristic peaks in Figure 2G corresponded to the negatively charged boron species, with peaks at 185.8 eV for B─Mg bond and 189.8 eV for B─B bond, confirming the successful preparation of the defect‐rich MgB_2_ MPs (≈1 µm, Figure S2, Supporting Information).

### 2.2 Functional Investigations of MgB_2_ MPs

**Figure 3:**
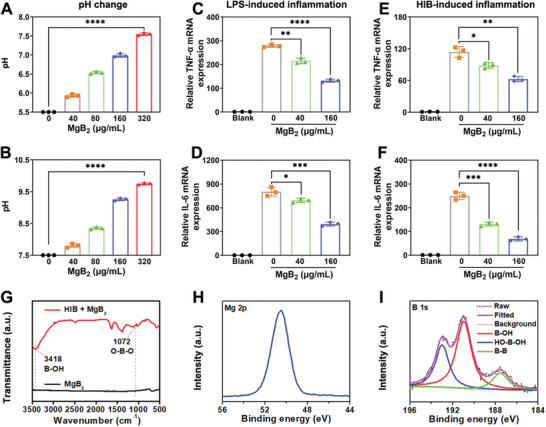
Functional characterization of MgB_2_ MPs. Changes of pH after MgB_2_ MPs with different concentrations hydrolysis under A) pH 5.5 or B) pH 7.5 solution. Expression of C) TNF‐α and D) IL‐6 in LPS induced Raw 264.7 cells treated with MgB_2_ MPs for 6 h via quantitative polymerase chain reaction (QPCR). Expression of E) TNF‐α and F) IL‐6 in HIB (HIB, heat inhibited MRSA) treated with MgB_2_ MPs for 6 h via QPCR. G) FT‐IR spectra of MgB_2_ MPs before and after incubation with HIB. XPS spectra of H) Mg 2p and I) B 1s orbitals for MgB_2_ MPs after incubation with HIB. Data represents mean ± SD, ^****^ *p* < 0.0001, ^***^ *p* < 0.001, ^**^ *p* < 0.01, ^*^ *p* < 0.05, n = 3.

To study the functions of MgB_2_ MPs, we assessed their ability to produce a mildly alkaline microenvironment. As depicted in **Figure** **3**A,B, the pH value increased with increasing concentrations of MgB_2_ MPs, indicating that hydrolysis of MgB_2_ MPs could generate the alkaline microenvironment. Moreover, MgB_2_ MPs were used to treat dead bacteria (HIB, heat inhibited MRSA) or LPS induced inflammation in vitro. Figure 3C,D showed that the expression of inflammatory molecules including interleukin 6 (IL‐6) and tumor necrosis factor α (TNF‐α) were induced by LPS, but they were dramatically inhibited by adding MgB_2_ MPs. Also, MgB_2_ MPs could inhibit HIB‐induced the expression of TNF‐α and IL‐6 (Figure 3E,F). These indicated that dead bacteria and LPS could cause an obvious inflammatory response in the immune cells. However, MgB_2_ MPs could interact with LPS or dead bacteria to inhibit the inflammatory response.

Fourier transform infrared spectroscopy (FT‐IR) spectra (Figure 3G) showed that the hydrolysate of MgB_2_ MPs generated a B─OH bond (at 3418 cm^−1^), and could react with HIB (heat inhibited bacteria, dead MRSA) to produce boronic ester O─B─O bond (1072 cm^−1^). Moreover, the XPS spectra revealed the characteristic peaks corresponding to Mg─B bond with a negative charge (185.8 eV in Figure 3H) and two boron species with a positive charge (192.7 eV for HO─B─OH bond and 190.9 eV for the B─OH bond in Figure 3I). These results collectively demonstrated that the boron hydroxyl groups, generated through the hydrolysis of MgB_2_ MPs, could act as the boron‐binding agent for capturing dead bacteria.

### 2.3 Antioxidative Ability and Biocompatibility of MgB_2_ MPs

The •OH and H_2_O_2_ were chosen as the typical reactive oxygen species (ROS) to assess the ROS eliminating properties of MgB_2_ MPs. In Figure S3A (Supporting Information), the 320 µg mL^−1^ MgB_2_ MPs could eliminate ≈6.6% of the H_2_O_2_. MgB_2_ MPs (320 µg mL^−1^) could quench more than 87% of •OH (Figure S3B, Supporting Information). Moreover, the antioxidative ability of MgB_2_ MPs was evaluated by the free radical scavenging test. Figure S3C (Supporting Information) shows that 320 µg mL^−1^ MgB_2_ MPs could scavenge 67% of the ABTS free radical. These results demonstrated that MgB_2_ MPs also could act as a ROS scavenging agent.

To consider the biocompatibility of MgB_2_ MPs, the cytotoxicity assay of MgB_2_ MPs was conducted prior to antibacterial treatment in vitro and in vivo. The cytotoxicity of MgB_2_ in vitro was evaluated by a lactate dehydrogenase (LDH) test. As shown in Figure S4 (Supporting Information), MgB_2_ MPs (160 µg mL^−1^) displayed the minimal cytotoxicity to HEK 293 cells in vitro. The toxicity of MgB_2_ MPs in vivo was also examined in Figure S5 (Supporting Information). Hematoxylin and eosin (H&E) staining images revealed no significant abnormalities or damage on following intravenous injection of MgB_2_ MPs for 14 days, suggesting the excellent biocompatibility of MgB_2_ MPs.

### 2.4 Antibacterial Performance of MgB_2_ MPs

**Figure 4:**
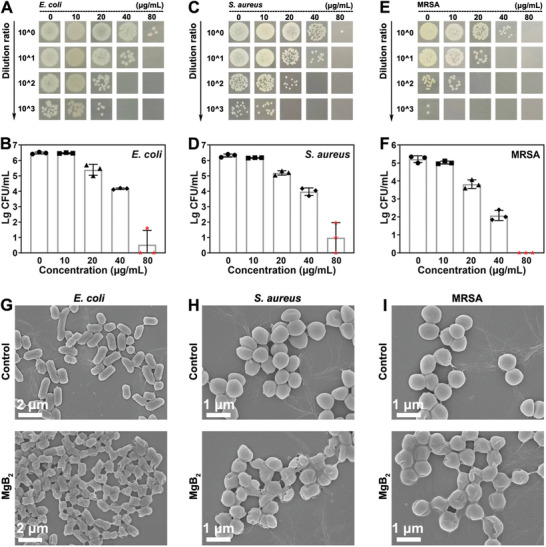
Antibacterial ability of MgB_2_ MPs. A) CFU counts of *E. coli* treated by MgB_2_ MPs. B) The inactivation efficiency of *E. coli* treated with MgB_2_ MPs. C) CFU counts of *S. aureus* treated by MgB_2_ MPs. D) The inactivation efficiency of *S. aureus* treated with MgB_2_ MPs. E) CFU counts of MRSA treated by MgB_2_ MPs. F) The inactivation efficiency of MRSA treated with MgB_2_ MPs. SEM images of G) *E. coli*, H) *S. aureus*, and I) MRSA subjected to MgB_2_ MPs for 60 min.

We evaluated the antibacterial performance of MgB_2_ MPs against three representative strains including *E. coli*, *S. aureus*, and MRSA. As depicted in **Figure** **4**A, the colony forming unit (CFU) counts of *E. coli* obviously decreased by increasing the concentrations of MgB_2_ MPs. Figure 4B revealed that 80 µg mL^−1^ MgB_2_ MPs could cause 5.9 log bacterial inactivation against *E. coli*. Similarly, the CFU count of *S. aureus* decreased after treatment with MgB_2_ MPs (Figure 4C), and the inactivation efficiency of 80 µg mL^−1^ MgB_2_ MPs for *S. aureus* was 5.3 log (Figure 4D). Furthermore, as shown in Figure 4E, MgB_2_ MPs (80 µg mL^−1^) could completely inhibit the growth of MRSA. The inactivation efficiency of 80 µg mL^−1^ MgB_2_ MPs against MRSA was 5.2 log, as shown in Figure 4F.

As the hydrolysis of MgB_2_ MPs could generate Mg^2+^ and an alkaline microenvironment, we studied the antibacterial performance of Mg^2+^ and different pH conditions. Figure S6 (Supporting Information) shows that 80 µg mL^−1^ Mg^2+^ had no obvious antibacterial activity. Figure S7 (Supporting Information) shows that 2 log bacteria inactivated under pH 10.5, while the bacteria maintained good activity under pH < 9.5. The result further proved that the local high alkaline microenvironment from the hydrolysis of MgB_2_ MPs could cause bacterial death in vitro.

Next, SEM was applied to observe the morphological changes of bacteria after treatment with MgB_2_ MPs. In the control group, *E. coli* displayed an intact rod‐like shape, whereas *E. coli* treated with MgB_2_ MPs showed an uneven and irregular morphology (Figure 4G). Similarly, *S. aureus* and MRSA in the absence of treatment maintained their spherical and smooth shape, whereas treatment with MgB_2_ MPs resulted in disrupted and wrinkled morphology for both *S. aureus* and MRSA, as observed in Figure 4H,I. These results proved that MgB_2_ MPs could kill MRSA, *E. coli*, and *S. aureus* by compromising the integrity of the bacterial structure.

### 2.5 Characterization and Property Studies of MgB_2_ MN

**Figure 5:**
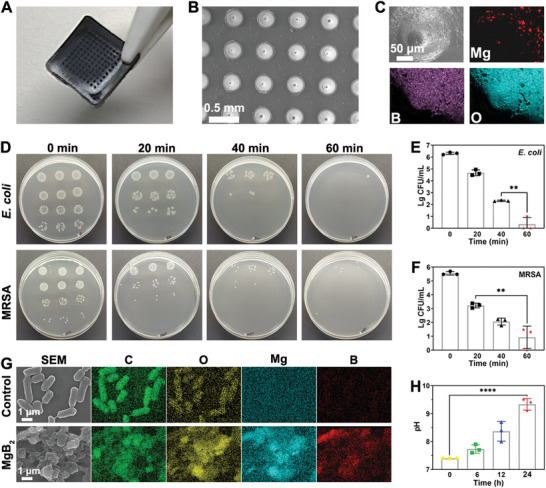
Characterization and property investigations of MgB_2_ MN. A) Photograph of MgB_2_ MN. B) SEM image of MgB_2_ MN. C) SEM and elemental mapping images of a single needle in MgB_2_ MN. D) CFU counts of bacteria treated by MgB_2_ MN over time. The inactivation efficiency of E) *E. coli* and F) MRSA after incubated with MgB_2_ MN. G) SEM and elemental mapping images of bacteria after treatment with MgB_2_ MN. H) Change of pH after MgB_2_ MN hydrolysis in bacterial solutions. Data represents mean ± SD, ^****^ *p* < 0.0001, ^**^ *p* < 0.01, *n* = 3.

We used the poly(dimethyl siloxane) (PDMS) mold to prepare MgB_2_ MN. As shown in **Figure** **5**A,B, the MgB_2_ MN exhibited a needle height of 600 µm and a base diameter of 9 mm. The distribution of Mg, B, and O elements in the MN was confirmed by the element mapping images, indicating the successful integration of MgB_2_ MPs (Figure 5C). The compression force of MgB_2_ MN was greater than 0.4 N per MN, which was sufficiently high to penetrate the skin (Figure S8, Supporting Information). The MgB_2_ MN penetrated the mouse wound, as evidenced by rhodamine staining. The penetration depth was 200 µm (Figure S9, Supporting Information). H&E staining of the extracted mouse skin tissue in Figure S10 (Supporting Information) proved that MgB_2_ MN also could insert the stratum corneum and penetrate into the epidermal layer.

Leveraging the antibacterial properties of MgB_2_ MPs, it was significant to study the antibacterial performance of MgB_2_ MNs. Figure S11 (Supporting Information) showed that the dosage of 320 µg mL^−1^ MgB_2_ MPs was optimized to prepare the MgB_2_ MNs for bacterial inactivation. Next, we further investigated the antibacterial effect of MgB_2_ MN against both *E. coli* and MRSA. Figure 5D shows that the bacterial counts for *E. coli* and MRSA gradually decreased over time following the treatment with MgB_2_ MN. After 1 h of treatment, MgB_2_ MN exhibited a remarkable bacterial inactivation efficiency of 5.9 log for *E. coli* (Figure 5E). Similarly, MgB_2_ MN caused a 4.6 log reduction in bacterial count for MRSA (Figure 5F). These findings unequivocally demonstrated the potent antibacterial efficacy of MgB_2_ MN against both *E. coli* and MRSA.

Considering the identified antimicrobial agents generated by MgB_2_ MPs hydrolysis, it was crucial to study the hydrolysis process of MgB_2_ MN during the antibacterial treatment. Figure 5G showed that the bacteria in the control group maintained their intact rod‐like morphology with only C and O elements, whereas the bacteria treated with the MgB_2_ MN group displayed irregular morphology with C, O, Mg, and B elements. Metal cations are known to disrupt bacterial membranes by modifying the permeability and membrane potential. These results clearly demonstrated that MgB_2_ MN effectively killed bacteria by compromising their structural integrity. Furthermore, the pH value increased with the time changes, indicating that MgB_2_ MN hydrolysis also could generate an alkaline microenvironment (Figure 5H). Thus, the bacterial death could be attributed to the local high alkaline condition produced by MgB_2_ MN hydrolysis owing to bacterial inactivation under the high pH condition. As shown in Figure S12A,B (Supporting Information), the viability of HaCat and 3T3 cells was obviously higher than that in the control group, indicating that MgB_2_ MN could promote the proliferation of fibroblasts and keratinocytes. Meanwhile, MgB_2_ MN accelerated the migration of HaCat and 3T3 cells (Figure S12C,D, Supporting Information). The results proved that MgB_2_ MN not only could kill bacteria, but also promoted the proliferation and migration of fibroblasts and keratinocytes.^[^ ^38^, ^39^ ^]^

### 2.6 The Dead Bacteria‐Trapping Ability of MgB_2_ MN

**Figure 6:**
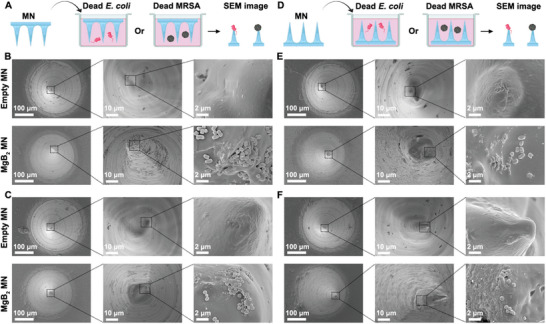
The dead bacteria‐trapping ability of MgB_2_ MN. A) Scheme of MN (needles down) for trapping dead bacteria. B) SEM image of empty MN (control) and MgB_2_ MN after incubation with dead *E. coli*. C) SEM image of empty MN (control) and MgB_2_ MN after incubation with dead MRSA. D) Scheme of MN (needles upward) for trapping dead bacteria. E) SEM image of empty MN (control) and MgB_2_ MN after incubation with dead *E. coli*. F) SEM image of empty MN (control) and MgB_2_ MN after incubation with dead MRSA.

The MgB_2_ MN trapping dead bacteria is required for their antibacterial treatment because dead bacteria‐induced inflammation would hamper wound healing. We used SEM to further study the dead bacteria‐trapping ability of MgB_2_ MN. Figure S13 (Supporting Information) shows that a single needle of MgB_2_ MN kept the original microneedle morphology after incubation without dead bacteria in saline. The needles downward first were incubated with dead bacteria in saline (**Figure** **6**A). After incubation with dead bacteria, many dead *E. coli* or MRSA were observed on the surface of a needle of MgB_2_ MN, but the empty MN could not trap dead bacteria (Figure 6B,C). Next, the needles upward were incubated with dead bacteria in saline (Figure 6D). After incubation for 24 h, SEM images showed that many dead *E. coli* or MRSA bound on the surface of a needle of MgB_2_ MN, but the empty MN could capture dead *E. coli* or MRSA (Figure 6E,F). Results proved that MgB_2_ MN could trap dead both MRSA and *E. coli*. Thus, it was necessary to use the MgB_2_ MN for trapping dead bacteria.

### 2.7 Treatment of Subcutaneous Abscess

**Figure 7:**
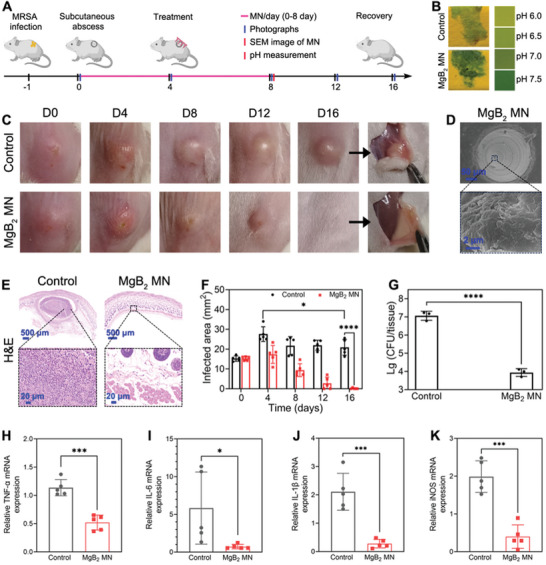
Treatment of subcutaneous abscess. A) Scheme of MgB_2_ MN for treating subcutaneous abscess. B) The pH values of infected tissues on the 8th day. C) Photographs of mice with subcutaneous abscesses for different days. D) SEM images of MgB_2_ MN after treating subcutaneous abscess on the 8th day. E) H&E staining photomicrographs of the infected tissues. F) The lesion areas of the infected site. G) Quantitative bacterial colonies from the infected tissues. Secretion level of H) TNF‐α, I) IL‐6, J) IL‐1β, and K) iNOS in infected tissues after 16 days of treatment. Data were based on mean ± SD (^***^ *p* < 0.0001, ^*^ *p* < 0.01, *n* = 3 in G, n = 5 in F and H–K).

We constructed MRSA infected subcutaneous abscess as a local bacterial infection model to assess the therapeutic effect of MgB_2_ MN in mice (**Figure** **7**A). In Figure 7B, after treatment of MgB_2_ MN for 8 days, the pH value of infected tissues was higher than that of control group, suggesting that MgB_2_ MN could regulate acidic microenvironment. The obvious remission was seen after treating with MgB_2_ MN for 16 days, but the severe ulceration and clear abscess could be observed in the control group (Figure 7C). SEM images in Figure 7D show that MgB_2_ MN could trap dead bacteria and remove these bacteria from the infected tissues. Figure 7E shows the much more neutrophils around the infected sites in the control group, whereas no obvious neutrophils could be observed in the MgB_2_ MN group. The area of MRSA infected site was 20.9 and 0.1 mm^2^ for the mice in the control and MgB_2_ MN group, respectively (Figure 7F). Moreover, the bacterial inactivation efficiency of MgB_2_ MN in vivo was studied by measuring the bacterial CFU number of infected tissues. Figure 7G showed the bacterial inactivation efficiency for the MgB_2_ MN group was 3.1 log. The TNF, IL‐6, IL‐1β, and iNOS signaling pathways were largely inhibited after MgB_2_ MN treatment, indicating that MgB_2_ MN had the ROS removing and anti‐inflammatory abilities against subcutaneous abscess in mice (Figure 7H–K). These results proved that MgB_2_ MN might kill bacteria and remove dead bacteria in vivo, reduced inflammation, and promote the recovery of subcutaneous abscess.

### 2.8 Treatment of Wound Infection

**Figure 8:**
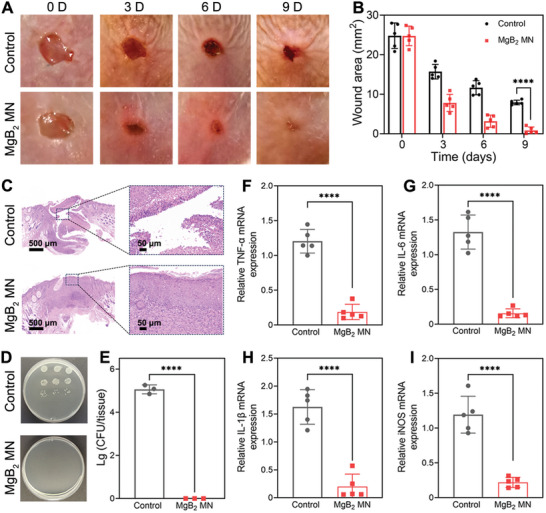
Treatment of MRSA infected wounds. A) Photographs of wounds on mice after treatment of different groups. B) Area changes of wounds treated by different groups of mice. C) H&E staining images of wound tissues. D) Photograph of CFU counts and E) quantitative bacterial CFU from wound tissues after 9 days of treatment. Secretion level of F) TNF‐α, G) IL‐6, H) IL‐1β, and I) iNOS from MRSA infected wound tissues after 9 days of treatment. Data were based on mean ± SD (^****^ *p* < 0.0001, *n* = 3 in E, *n* = 5 in B and F–I).

MRSA infected wounds was fabricated to further validate the therapeutic effect of MgB_2_ MN in vivo. After 9‐day treatment, **Figure** **8**A showed no obvious lesions in the MgB_2_ MN group, whereas the clear inflammation and scab were observed in the control group. In Figure 8B, the healing rate of MRSA infected wounds in the MgB_2_ MN group was obviously higher than that in the control group. In addition, histological analysis was further applied to assess the wound healing state. Results showed that MgB_2_ MN could dramatically accelerate wound healing (Figure 8C). As shown in Figure 8D,E, MgB_2_ MN caused 5 log bacterial inactivation with comparison of control group. Notably, the MgB_2_ MN treatment inhibited the expression of inflammatory TNF‐α, IL‐1β, IL‐6, and iNOS signaling pathways in wounds, indicating that MgB_2_ MN could reduce the inflammation against infected wounds in mice (Figure 8F–I). Results certified that the MgB_2_ MN also could treat wound infection.

### 2.9 Treatment of Dead Bacteria‐Induced Wound Inflammation

Another significant therapeutic effect of MgB_2_ MN is its ability to eliminate dead bacteria and reduce excessive inflammation. To evaluate the effect, we used a mouse model of HIB‐induced wound inflammation. As shown in Figure S14A (Supporting Information), dead bacteria caused noticeable swelling and redness of wounds, which could be obviously inhibited by using MgB_2_ MN. Next, the wound areas were dissected and stained by H&E and immunofluorescence tests to further evaluate the histopathological changes and inflammatory conditions. H&E staining images showed that the epidermis of HIB‐treated wounds without clear pathological changes after treatment of MgB_2_ MN. Comparatively, the hematoxylin‐positive and lipogenesis cells were distinctly increased in the dermis with dead bacteria, which were inhibited by MgB_2_ MN. These findings indicated that MgB_2_ MN could reduce dead bacteria‐induced inflammation (Figure S14B, Supporting Information). Furthermore, we accomplished the immunofluorescence staining experiment using the neutrophil cell marker myeloperoxidase (MPO) and the macrophage cell marker F4/80. Consistently, the immunofluorescence staining demonstrated that MgB_2_ MN dramatically decreased the infiltration of dead bacteria‐induced macrophages (F4/80‐positive cells) and neutrophils (MPO‐positive cells) (Figure S14C,D, Supporting Information). Taken together, these findings proved that dead bacteria could cause serious wound inflammation, but MgB_2_ MN could effectively inhibit the dead bacteria‐induced wound inflammation.

## 3 Conclusion

In summary, we have introduced a novel reactive microneedle (MgB_2_ MN) patch that regulated the acidic microenvironment in bacteria infected both subcutaneous abscesses and wounds, killed bacteria, and removed dead bacteria to avoid local inflammation for wound healing. Specifically, MgB_2_ MPs were prepared by a simple top‐down method. In vitro results proved that MgB_2_ MPs not only achieved broad antibacterial effects against *E. coli*, *S. aureus*, and MRSA but also could inhibit the inflammatory response against HIB‐induced inflammation. Also, MgB_2_ MPs integrated MNs could kill bacteria and trap the dead bacteria. In vitro and in vivo results proved that MgB_2_ MNs inhibited bacterial survival, eliminated dead bacteria, and decreased the dead bacteria excessive inflammation. Taken together, the MgB_2_ MNs patch could realize efficient treatment of bacterial skin infections and dead bacteria‐induced wound inflammation. This study provides a promising avenue for regulating bacteria‐infected microenvironments and eliminating dead bacteria to treat skin infections and inflammation.

## Conflict of Interest

The authors declare no conflict of interest.

## Author Contributions

J.Y.S. and Y.J.Z. conceived the idea; J.Y.S. conducted the experiments and wrote the manuscript; J.Y.S., X.Y.W., J.Y.C., and J.J.G. reviewed and commented in the manuscript.

## Supporting information

Supporting Information

## Data Availability

The data that support the findings of this study are available from the corresponding author upon reasonable request.

## References

[1] A. B. Raff , D. Kroshinsky , J. Am. Med. Assoc. 2016, 316, 325.

[2] C. Youn , N. K. Archer , L. S. Miller , J. Invest. Dermatol. 2020, 140, 1488. 32407714 10.1016/j.jid.2020.04.012 PMC7387158

[3] R. S. Daum , L. G. Miller , L. Immergluck , S. Fritz , C. B. Creech , D. Young , N. Kumar , M. Downing , S. Pettibone , R. Hoagland , S. J. Eells , M. G. Boyle , T. C. Parker , H. F. Chambers , D. Team , N. Engl. J. Med. 2017, 376, 2545. 28657870 10.1056/NEJMoa1607033 PMC6886470

[4] T. Nakatsuji , T. R. Hata , Y. Tong , J. Y. Cheng , F. Shafiq , A. M. Butcher , S. S. Salem , S. L. Brinton , A. K. Rudman Spergel , K. Johnson , B. Jepson , A. Calatroni , G. David , M. Ramirez‐Gama , P. Taylor , D. Y. M. Leung , R. L. Gallo , Nat. Med. 2021, 27, 700. 33619370 10.1038/s41591-021-01256-2 PMC8052297

[5] V. Choi , J. L. Rohn , P. Stoodley , D. Carugo , E. Stride , Nat. Rev. Microbiol. 2023, 21, 555. 37258686 10.1038/s41579-023-00905-2

[6] S. A. Eming , P. Martin , M. Tomic‐Canic , Sci. Transl. Med. 2014, 6, 265sr6. 25473038 10.1126/scitranslmed.3009337 PMC4973620

[7] A. Gupta , S. Mumtaz , C. H. Li , I. Hussain , V. M. Rotello , Chem. Soc. Rev. 2019, 48, 415. 30462112 10.1039/c7cs00748e PMC6340759

[8] S. E. Rossiter , M. H. Fletcher , W. M. Wuest , Chem. Rev. 2017, 117, 12415. 28953368 10.1021/acs.chemrev.7b00283 PMC5869711

[9] S. Eckhardt , P. S. Brunetto , J. Gagnon , M. Priebe , B. Giese , K. M. Fromm , Chem. Rev. 2013, 113, 4708. 23488929 10.1021/cr300288v

[10] M. R. Reithofer , A. Lakshmanan , A. T. Ping , J. M. Chin , C. A. Hauser , Biomaterials 2014, 35, 7535. 24933510 10.1016/j.biomaterials.2014.04.102

[11] Z. Liu , K. Guo , L. Yan , K. Zhang , Y. Wang , X. Ding , N. Zhao , F. J. Xu , Nat. Commun. 2023, 14, 5132. 37612285 10.1038/s41467-023-40830-9 PMC10447547

[12] L. Jin , F. Cao , Y. Gao , C. Zhang , Z. Qian , J. Zhang , Z. Mao , Adv. Mater. 2023, 35, 2301349. 10.1002/adma.202301349 37083074

[13] J. Shan , J. Che , C. Song , Y. Zhao , Smart Med. 2023, 2, 20220025. 10.1002/SMMD.20220025 PMC11235951 39188347

[14] W. Xiu , L. Ren , H. Xiao , Y. Zhang , D. Wang , K. Yang , S. Wang , L. Yuwen , X. Li , H. Dong , Q. Li , Y. Mou , Y. Zhang , Z. Yin , B. Liang , Y. Gao , L. Wang , Sci. Adv. 2023, 9, ade5446. 10.1126/sciadv.ade5446 PMC12164730 36696490

[15] Z. Su , L. Kong , Y. Dai , J. Tang , J. Mei , Z. Qian , Y. Ma , Q. Li , S. Ju , J. Wang , W. Fan , C. Zhu , Sci. Adv. 2022, 8, abn1701. 10.1126/sciadv.abn1701 PMC8993125 35394829

[16] J. Ouyang , X. Ji , X. Zhang , C. Feng , Z. Tang , N. Kong , A. Xie , J. Wang , X. Sui , L. Deng , Y. Liu , J. S. Kim , Y. Cao , W. Tao , Proc. Natl. Acad. Sci. USA 2020, 117, 28667. 33139557 10.1073/pnas.2016268117 PMC7682336

[17] G. Qing , X. Zhao , N. Gong , J. Chen , X. Li , Y. Gan , Y. Wang , Z. Zhang , Y. Zhang , W. Guo , Y. Luo , X. J. Liang , Nat. Commun. 2019, 10, 4336. 31551496 10.1038/s41467-019-12313-3 PMC6760232

[18] Y. Meng , L. Chen , Y. Chen , J. Shi , Z. Zhang , Y. Wang , F. Wu , X. Jiang , W. Yang , L. Zhang , C. Wang , X. Meng , Y. Wu , W. Bu , Nat. Commun. 2022, 13, 7353. 36446788 10.1038/s41467-022-35050-6 PMC9708144

[19] Y. Sang , W. Li , H. Liu , L. Zhang , H. Wang , Z. Liu , J. Ren , X. Qu , Adv. Funct. Mater. 2019, 29, 1900518.

[20] H. Nishino , T. Fujita , A. Yamamoto , T. Fujimori , A. Fujino , S.‐I. Ito , J. Nakamura , H. Hosono , T. Kondo , J. Phys. Chem. C 2017, 121, 10587.

[21] M. Fan , Y. Wen , D. Ye , Z. Jin , P. Zhao , D. Chen , X. Lu , Q. He , Adv. Healthcare Mater. 2019, 8, 1900157. 10.1002/adhm.201900157 30968583

[22] S. Li , H. Gunda , K. G. Ray , C. S. Wong , P. Xiao , R. W. Friddle , Y. S. Liu , S. Kang , C. Dun , J. D. Sugar , R. D. Kolasinski , L. F. Wan , A. A. Baker , J. R. I. Lee , J. J. Urban , K. Jasuja , M. D. Allendorf , V. Stavila , B. C. Wood , Nat. Commun. 2021, 12, 6268. 34725350 10.1038/s41467-021-26512-4 PMC8560812

[23] H. Dong , Q. Xiang , Y. Gu , Z. Wang , N. G. Paterson , P. J. Stansfeld , C. He , Y. Zhang , W. Wang , C. Dong , Nature 2014, 511, 52. 24990744 10.1038/nature13464

[24] J. A. Peters , Coord. Chem. Rev. 2014, 268, 1.

[25] V. Sampath , Agric. Nat. Resour. 2018, 52, 115.

[26] G. Springsteen , B. Wang , Tetrahedron 2002, 58, 5291.

[27] Y. Tian , Y. Qi , Y. Fang , Z. Xu , L. Sun , Y. Dong , G. Ning , J. Ye , ACS Appl. Bio Mater. 2023, 6, 2837. 10.1021/acsabm.3c00290 37319103

[28] H. Gunda , L. E. Klebanoff , P. A. Sharma , A. K. Varma , V. Dolia , K. Jasuja , V. Stavila , ACS Mater. Lett. 2021, 3, 535.

[29] R. Kucukosman , Z. Isik , K. Ocakoglu , N. Dizge , S. Ozdemir , M. S. Yalcin , P. Sharma , D. Balakrishnan , Chemosphere 2023, 339, 139340. 37379977 10.1016/j.chemosphere.2023.139340

[30] Z. Wang , J. Luan , A. Seth , L. Liu , M. You , P. Gupta , P. Rathi , Y. Wang , S. Cao , Q. Jiang , X. Zhang , R. Gupta , Q. Zhou , J. J. Morrissey , E. L. Scheller , J. S. Rudra , S. Singamaneni , Nat. Biomed. Eng. 2021, 5, 64. 33483710 10.1038/s41551-020-00672-y PMC8020465

[31] J. Yu , J. Wang , Y. Zhang , G. Chen , W. Mao , Y. Ye , A. R. Kahkoska , J. B. Buse , R. Langer , Z. Gu , Nat. Biomed. Eng. 2020, 4, 499. 32015407 10.1038/s41551-019-0508-y PMC7231631

[32] Z. Chen , H. Li , Y. Bian , Z. Wang , G. Chen , X. Zhang , Y. Miao , D. Wen , J. Wang , G. Wan , Y. Zeng , P. Abdou , J. Fang , S. Li , C. J. Sun , Z. Gu , Nat. Nanotechnol. 2021, 16, 933. 33972760 10.1038/s41565-021-00910-7

[33] W. Chen , J. Wainer , S. W. Ryoo , X. Qi , R. Chang , J. Li , S. H. Lee , S. Min , A. Wentworth , J. E. Collins , S. Tamang , K. Ishida , A. Hayward , R. Langer , G. Traverso , Sci. Adv. 2022, 8, abk1792. 10.1126/sciadv.abk1792 PMC8730401 34985942

[34] K. J. McHugh , L. Jing , S. Y. Severt , M. Cruz , M. Sarmadi , H. S. N. Jayawardena , C. F. Perkinson , F. Larusson , S. Rose , S. Tomasic , T. Graf , S. Y. Tzeng , J. L. Sugarman , D. Vlasic , M. Peters , N. Peterson , L. Wood , W. Tang , J. Yeom , J. Collins , P. A. Welkhoff , A. Karchin , M. Tse , M. Gao , M. G. Bawendi , R. Langer , A. Jaklenec , Sci. Transl. Med. 2019, 11, aay7162. 10.1126/scitranslmed.aay7162 PMC7532118 31852802

[35] Y. Sun , J. Liu , H. Wang , S. Li , X. Pan , B. Xu , H. Yang , Q. Wu , W. Li , X. Su , Z. Huang , X. Guo , H. Liu , Adv. Funct. Mater. 2021, 31, 2100218.

[36] R. Jamaledin , C. K. Y. Yiu , E. N. Zare , L. N. Niu , R. Vecchione , G. Chen , Z. Gu , F. R. Tay , P. Makvandi , Adv. Mater. 2020, 32, 2002129. 10.1002/adma.202002129 32602146

[37] Y. Xiang , J. Lu , C. Mao , Y. Zhu , C. Wang , J. Wu , X. Liu , S. Wu , K. Y. H. Kwan , K. M. C. Cheung , K. W. K. Yeung , Sci. Adv. 2023, 9, adf0854. 10.1126/sciadv.adf0854 PMC9995069 36888703

[38] J. R. Sharpe , K. L. Harris , K. Jubin , N. J. Bainbridge , N. R. Jordan , Br. J. Dermatol. 2009, 161, 671. 19438462 10.1111/j.1365-2133.2009.09168.x

[39] T. Cui , J. Yu , C. F. Wang , S. Chen , Q. Li , K. Guo , R. Qing , G. Wang , J. Ren , Adv. Sci. 2022, 9, 2201254. 10.1002/advs.202201254 PMC9353480 35596608

