# Supplementary material for: Reactive Microneedle Patches with Antibacterial and Dead Bacteria‐Trapping Abilities for Skin Infection Treatment
Source: Adv Sci (Weinh). 2024 Apr 6;11(23):2309622. doi: 10.1002/advs.202309622 (PMC11186059; doi:10.1002/advs.202309622)
Supplement: Supplementary file 1 — Supporting Information [file ADVS-11-2309622-s001.pdf]

ADVANCED SCIENCE
Open Access

# Supporting Information

for *Adv. Sci.*, DOI 10.1002/advs.202309622

Reactive Microneedle Patches with Antibacterial and Dead Bacteria-Trapping Abilities for Skin Infection Treatment

*Jingyang Shan, Xiangyi Wu, Junyi Che, Jingjing Gan and Yuanjin Zhao**

# Supporting Information

## Reactive Microneedle Patches with Antibacterial and Dead Bacteria-Trapping Abilities for Skin Infection Treatment

Jingyang Shan, Xiangyi Wu, Junyi Che, Jingjing Gan, Yuanjin Zhao*

J. Shan, X. Wu, J. Che, J. Gan, Y. Zhao

Department of Rheumatology and Immunology

Nanjing Drum Tower Hospital

School of Biological Science and Medical Engineering

Southeast University

Nanjing 210096, China

Email: yjzhao@seu.edu.cn

J. Shan

Key Laboratory of Organic Electronics and Information Displays

Jiangsu Key Laboratory for Biosensors

Institute of Advanced Materials (IAM)

Nanjing University of Posts and Telecommunications

Nanjing 210023, China

**Supplementary Information:**

**Supplementary Methods**

**Supplementary Figures S1-S14**

# Supplementary Methods

## 1.1. Chemical reagents

All chemical reagents were analytical grade and directly used in this work without further purification. Magnesium Boron ($MgB_2$, 99.9%, AR) powder was purchased from Aladdin (Shanghai, China). Lactate dehyrogenase (LDH)-cytotoxicity colorimetric assay kit, HaCat cell, 3T3 cell, cck-8, and HEK 293 cell were bought from KeyGen (Nanjing, China). Ultrapure water (Millipore, 18.2 MΩ) was used in all experiments.

## 1.2. Preparation of $MgB_2$ MPs

$MgB_2$ powder (100 mg) was dispersed into 40 mL of NMP solution and sonicated in the water bath for 12 h. $MgB_2$ MPs were purified by centrifugation of 5000 rpm for 10 min and washed for 3 times using ethanol.

## 1.3. $H_2O_2$ scavenging test

$H_2O_2$ can react with $Ti(SO_4)_2$ to generate the yellow complex with a characteristic absorption peak at 405 nm. $H_2O_2$ (100 μL, 0.3 M) was incubated with $MgB_2$ MPs (100 μL, final concentration: 80, 160, and 320 μg/mL) at 37°C for 5 h. The 150 μL supernatant solution was collected by removing $MgB_2$ MPs and then mixed with 50 μL $Ti(SO_4)_2$ solution (6 mg/mL). Their absorbance was measured at the wavelength of 405 nm using the microplate reader.

## 1.4. •OH scavenging test

•OH scavenging activity test was studied by using salicylic acid. The salicylic acid can react with •OH to form the pink complex with a characteristic absorption at 510 nm. The work solutions containing $H_2O_2$ (100 μL, 50 mM) and $FeSO_4$ (2 mM, 100 μL) were co-incubated for 10 min. $MgB_2$ MPs (final concentration: 80, 160, and 320 μg/mL) were added into the mixed work reaction and co-incubated for 15 min. The salicylic acid (4 mM, 100 μL) was added into the above solution (100 μL) after

removing $MgB_2$ MPs. Their absorbance was measured at the wavelength of 510 nm using the microplate reader.

## 1.5. ABTS free radical scavenging test

The 2,2′-azino-bis(3-ethylbenzothiazoline 6-sulfonate) (ABTS) free radical scavenging ability of $MgB_2$ MPs was studied based on ABTS free radical scavenging assay kit (Beyotime Biotechnology, Shanghai, China). $MgB_2$ MPs (final concentration: 80, 160, and 320 μg/mL) were added to the working solution. After co-incubation for 10 min in the dark, the absorbance was measured at 734 nm by the microplate reader.

## 1.6. The pH variation of $MgB_2$ MPs hydrolysate

The 3 mL $MgB_2$ MPs solution (0-320 μg/mL) containing sodium citrate/citric acid buffer (pH 7.5 or 5.5) and performed in the shaker (200 rpm) at 37°C. After 24 h, the pH values of various solutions were detected by a pH meter.

## 1.7. Cytotoxicity assay

The cytotoxicity of $MgB_2$ MPs was evaluated using HEK293 cells. HEK293 cells were incubated with $MgB_2$ MPs (final concentration: 0-160 μg/mL) in the 96-well plate. Their cytotoxicity was evaluated after incubation at 37°C for 24 h by a LDH assay.

## 1.8. Bacterial culture

*S. aureus* (ATCC25923), MRSA (ATCC43300), and *E. coli* (ATCC25922) were used to investigate the antibacterial abilities of $MgB_2$ MPs and MNs.

## 1.9. Anti-inflammatory ability of $MgB_2$ MPs in vitro

LPS-induced inflammation: RAW264.7 ($2 \times 10^5$ cells) was seeded into the 12-well plate and incubated at 37°C overnight, and then added with PBS, LPS (200 ng/mL), or LPS + $MgB_2$ MPs (final concentration: 40 and 160 μg/mL) for 24 h. The $10^9$ CFU/mL MRSA were completely killed at 70°C in saline for 1 h to prepare HIB.

HIB-induced inflammation: RAW264.7 ($2 \times 10^5$ cells) was seeded into the 12-well plate and incubated at 37°C overnight, and then added with PBS, HIB ($10^8$ CFU/mL), or HIB + $MgB_2$ MPs (final concentration: 40 and 160 μg/mL) for 24 h. The total RNA was separated by an Eastep Super RNA isolation kit (Nanjing Vazyme, Rc112). The levels of IL-6 and TNF-α were measured via QPCR.

### 1.10. Antibacterial experiment

The antibacterial activities of $MgB_2$ MPs were studied by CFU counting method. The bacteria (*S. aureus*, MRSA, or *E. coli*; $2 \times 10^6$ CFU/mL; 100 μL) were incubated with $MgB_2$ MPs (0-80 μg/mL, 100 μL). After 1 h, the ten microliters of bacterial suspensions were serially diluted with sterile saline and grew on the LB agar plate, then CFU were counted.

### 1.11. Fabrication of $MgB_2$ MN

The $MgB_2$ MN was established using uniform silicone molds (Taizhou, China). The 2-hydroxy-2-methylpropiophenone (10 μL), polyvinylpyrrolidone aqueous solution (25%, 1 mL), and poly (ethylene glycol) diacrylate (200 μL) were mixed at room temperature as the MN solution. The MN solution (500 μL) was mixed with $MgB_2$ MPs (640 μg/mL, 500 μL) as $MgB_2$ MN solution. Then, 150 μL $MgB_2$ MN solutions were added into mold and filled for 10 min in the vacuum, and was cured using UV light irradiation for 15 s. $MgB_2$ MN patches were collected by demolding from MN molds.

### 1.12. Antibacterial performance of $MgB_2$ MN

The antibacterial performance of $MgB_2$ MN was studied by the CFU counting method. The bacterial suspensions ($10^6$ CFU/mL, 200 μL) containing $MgB_2$ MN were incubated in a shaker (shaking speed: 200 rpm) at 37°C. At regular intervals, the ten microliters of bacterial suspensions were serially diluted using saline and plated on LB agar plates. Finally, the number of colonies were counted.

### 1.13. The pH variation of $MgB_2$ MN hydrolysate

Sodium citrate/citric acid buffer solutions containing $MgB_2$ MNs (pH 7.5, 3 mL) and performed in the shaker (200 rpm) at 37°C. At regular intervals, their pH values were monitored using the pH meter.

### 1.14. Dead bacteria-trapping ability of $MgB_2$ MN

The dead bacteria ($10^8$ CFU/mL, 1 mL) containing $MgB_2$ MN were incubated at 37°C for 24 h. MNs were fixed with glutaraldehyde (2.5%) for 12 h at 4°C, then were performed sequential dehydrating treatments using the gradient ethanol solutions. The empty MNs without $MgB_2$ MPs as the control. The final MNs were observed by SEM after sputter-coating 5 nm gold.

### 1.15. Mechanical strength of $MgB_2$ MN

The force sensor was fastened above the fixed station and approached the $MgB_2$ MN at the speed of 0.2 mm/min. The force measurements began when the sensor first touched the MN tips and ended when the sensor traveled 0.2 mm.

### 1.16. Wound and skin insertion test

The rhodamine-stained $MgB_2$ MNs were inserted into the living mice skin and wound for 5 min to examine their penetration capability. MN insertion sites in wounds were observed by using an Olympus FV3000 confocal laser scanning microscope (CLSM). MN insertion sites in skin were observed by Hematoxylin and eosin (H&E) staining.

### 1.17. Cell proliferation assay

The keratinocytes (HaCat) and fibroblasts (3T3) were used in the proliferation assay. HaCat and 3T3 cells ($10^5$ cells) were incubated with $MgB_2$ MNs at 37°C for 48 h. The cell viability was evaluated by using a cck-8 assay.

### 1.18. Cell migration assay

The migration of fibroblasts and keratinocytes was studied by using the scratch assay. HaCat and 3T3 cells were plated into a 6-well plate to reach confluence, and then were scratched by $MgB_2$ MN and the empty MN (control). The images of cells were taken at 0 and 48 h using an Olympus inverted microscope.

### 1.19. Subcutaneous abscess model

All animals were handled in accordance with the Guidelines for the Protection and Use of Laboratory Animals of the National Institutes of Health and with the approval from the Ethical Review Committee of Nanjing Drum Tower Hospital. The animal experiments were conducted with the Animal Investigation Ethics Committee of The Affiliated Drum Tower Hospital of Nanjing University Medical School (2023AE02009).

The female Balb/c mice (18-22 g, Qinglong Mountain Company, China) were used to build the infected subcutaneous abscess model. We removed the dorsal hair of the mice. The 100 μL MRSA (OD = 1) were inoculated into the right side of spine on the back. After 1 day, the ten mice were randomly divided into two groups. These patches were inserted into MRSA infected subcutaneous abscess by hand and remained for 24 h. The subcutaneous abscess was treated with MNs on 8 days. At 8-day treatment, the bacteria infected skin tissues were harvested for pH measurement. At 16-day treatment, the bacteria infected skin tissues were harvested for histological analysis, counting bacterial CFU, and then the levels of TNF-α, iNOS, IL-1β, and IL-6 were measured via QPCR.

### 1.20. Wound infection model

The round wounds (~6 mm) were constructed and then infected ($10^7$ CFU MRSA) on the back of mice, all infected wounds were finally covered with a special kind of plastic sticker (Tegaderm Film, 3M, 1624W). After 24 h, the ten mice were randomly divided into two groups. These patches were inserted into MRSA infected wounds using our hand and remained on wounds for 24 h. All wounds were treated with MNs on 3 days. On day 9, the wounded skin was harvested for counting bacterial CFU, histological analysis, and then the levels of TNF-α, iNOS, IL-1β, and IL-6 were measured via QPCR.

### 1.21. Dead bacteria induced wound inflammation

The round wounds (~6 mm) were constructed and then infected ($10^7$ CFU HIB) on the back of mice, all infected wounds were finally covered with a special kind of plastic

sticker (Tegaderm Film, 3M, 1624W). After 1 day, the 10 mice were randomly divided into 2 groups. The pure wounds without infection in other ten mice were used as the control groups. The HIB-induced wounds showed an acute exudative state before MNs treatment. The pure wounds showed a crusted and dry state before MNs treatment. The wounds were treated with MNs on 3 days. On day 12, the wounded skin was harvested for the histological analysis.

### 1.22. Histological analysis

The infected tissues or major organs (heart, liver, spleen, lung and kidney) were dissected, fixed in 4% paraformaldehyde solution, paraffined, sectioned, and then analyzed by H&E staining.

### 1.23. Reproducibility and statistical analysis

All quantitative data in each experiment were repeated and evaluated from three independent experiments with similar results. The data were performed as mean ± SD. Statistical significance between groups was analyzed by one- or two-way ANOVA with a Tukey's post-hoc test for two groups in the GraphPad software (version 9.0.0), and the threshold for statistical significance was $P$ value < 0.05. Sample size (n) = 3 in Figures 3A-F, 5E, 5F, 5H, 7G, 8E, S6B, and S12A-B for statistical analysis. Sample size (n) = 5 in Figures 7F, 7H-K, and 8F-I for statistical analysis.

## Supplementary Figures

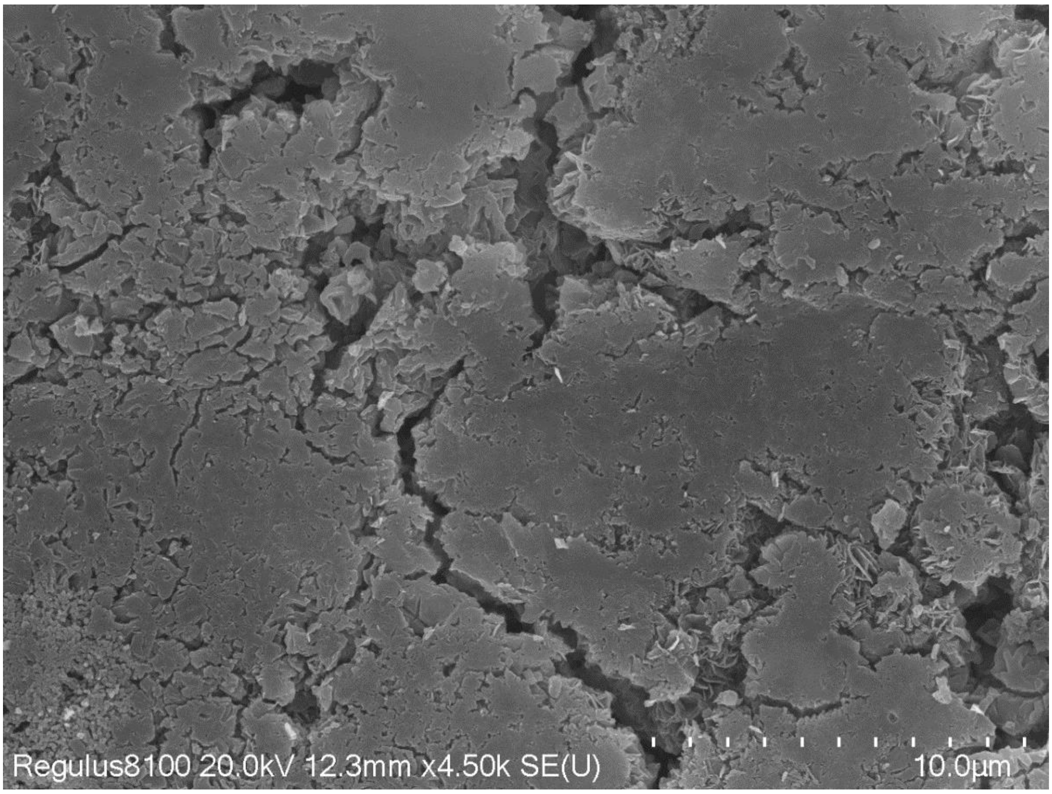


**Figure S1.** SEM image of bulk $MgB_2$ materials.

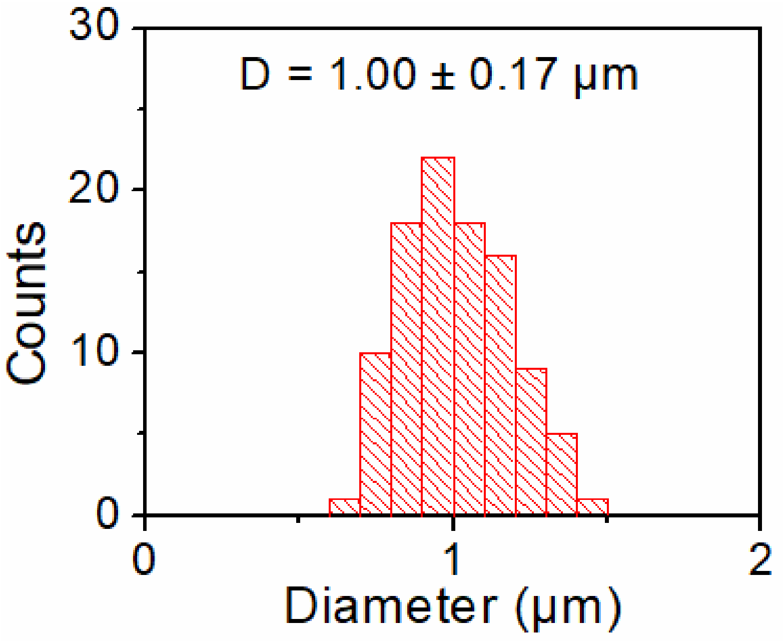


**Figure S2.** Size distribution of $MgB_2$ MPs.

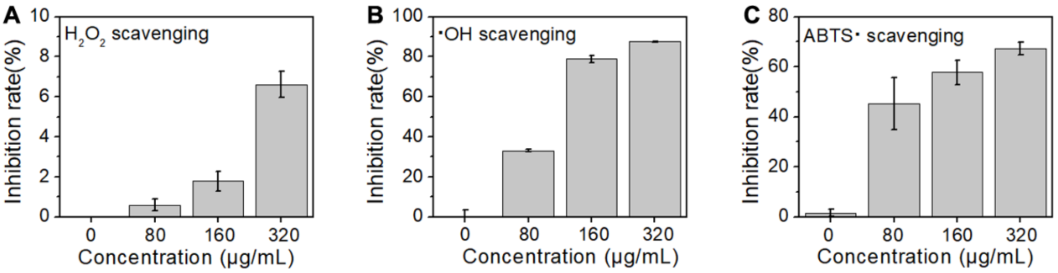


**Figure S3.** Reactive oxygen species (ROS) scavenging abilities of $MgB_2$ MPs: (A) $H_2O_2$, (B) •OH, and (C) ABTS•.

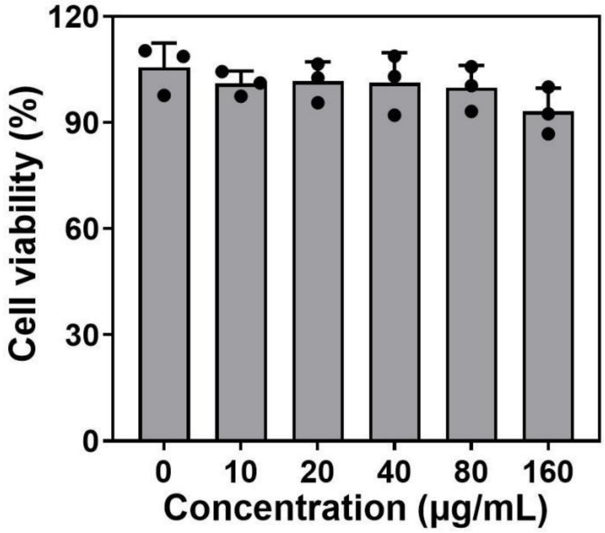


**Figure S4.** Cell viability of $MgB_2$ MPs.

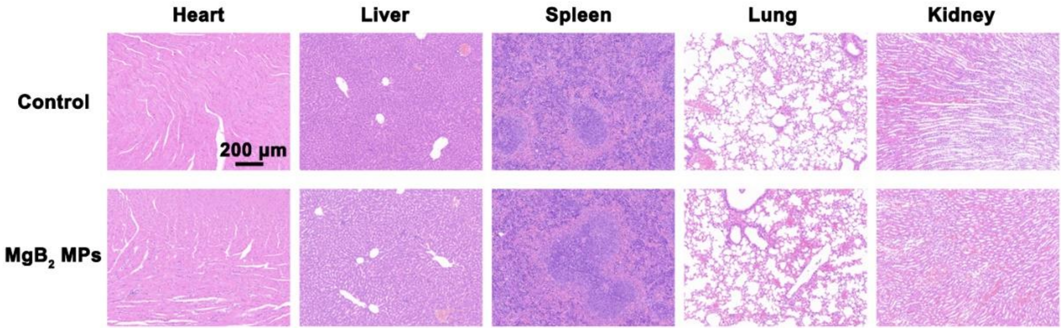


**Figure S5.** Biocompatibility of $MgB_2$ MPs in vivo.

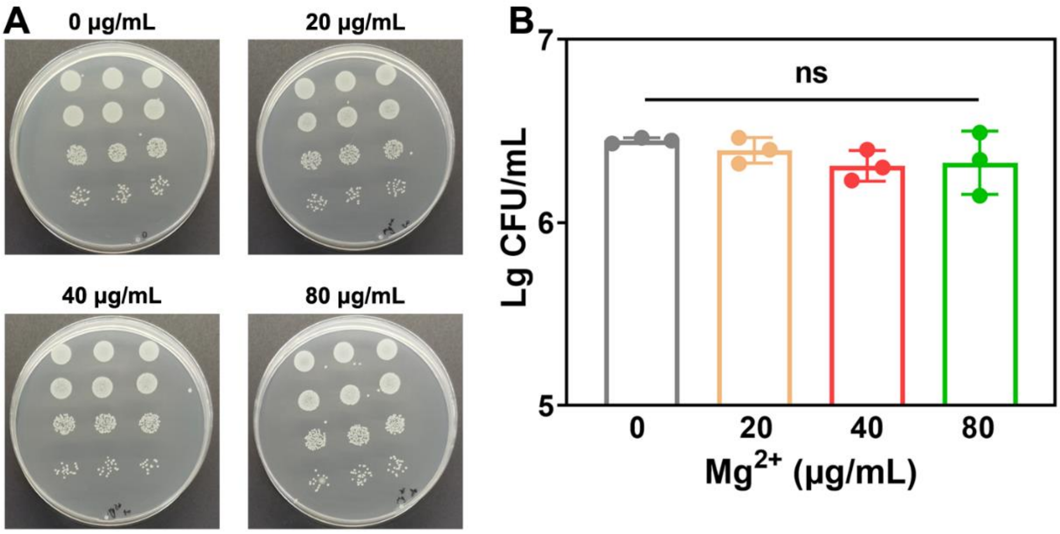


**Figure S6.** (A) CFU counts and (B) the inactivation efficiency of MRSA treated by $Mg^{2+}$ with different concentrations for 1 h.

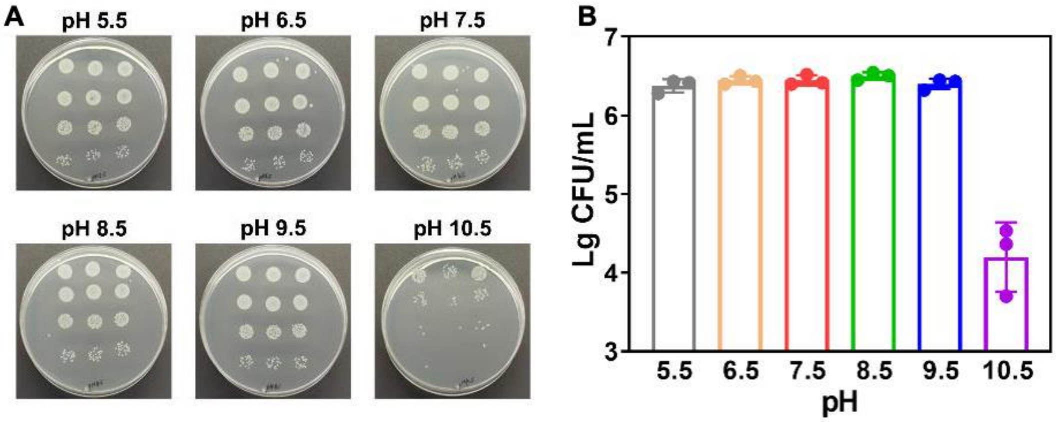


**Figure S7.** (A) CFU counts and (B) the inactivation efficiency of MRSA after incubation under different pH condition for 1 h.

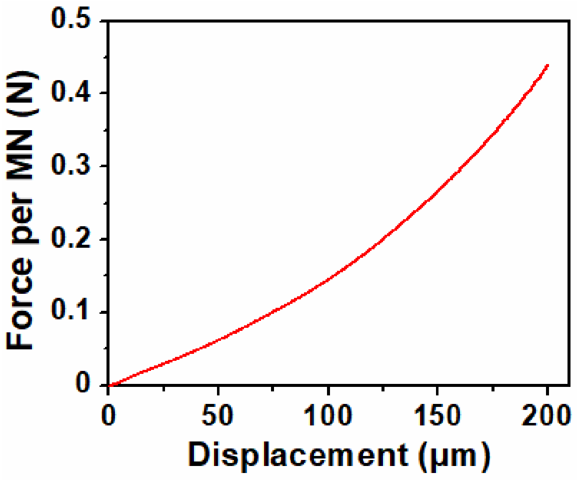


**Figure S8.** Mechanical property of the $MgB_2$ MN under normal compressive load.

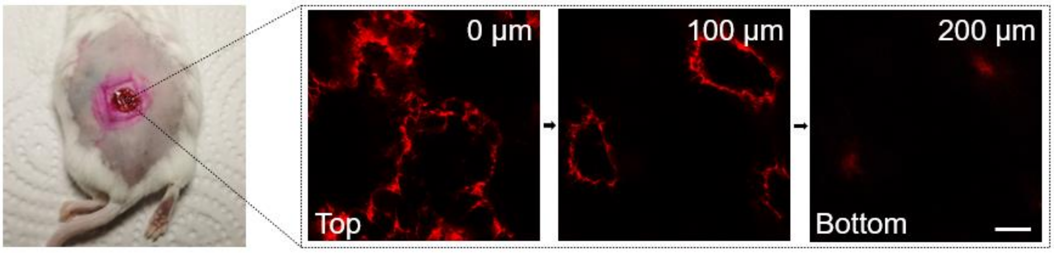


**Figure S9.** Left: mouse wound administered with the rhodamine-stained $MgB_2$ MN. Right: CLSM images of rhodamine staining, showing the different depths (0, 100, and 200 μm) caused by the penetration of $MgB_2$ MN on mouse wound. Scale bar, 200 μm.

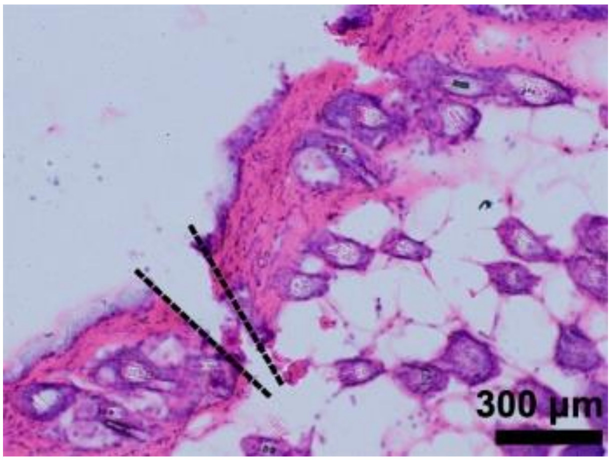


**Figure S10.** H&E-stained section of mouse skin showing the indents caused by the penetration of a single microneedle.

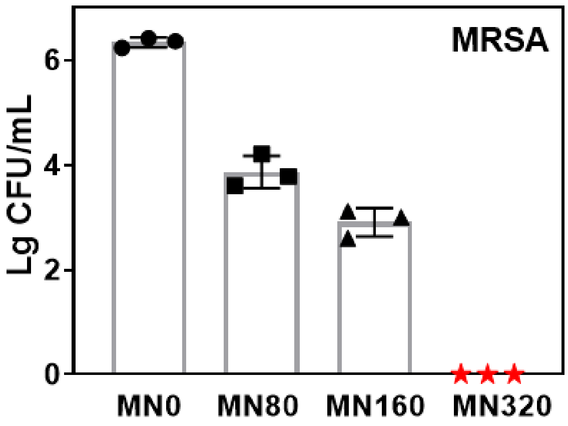


**Figure S11.** The inactivation efficiency of MRSA after incubation with different MNs. MN0, MN80, MN160, and MN320 were prepared by using $MgB_2$ MPs with the final concentration of 0, 80, 160, and 320 μg/mL, respectively.

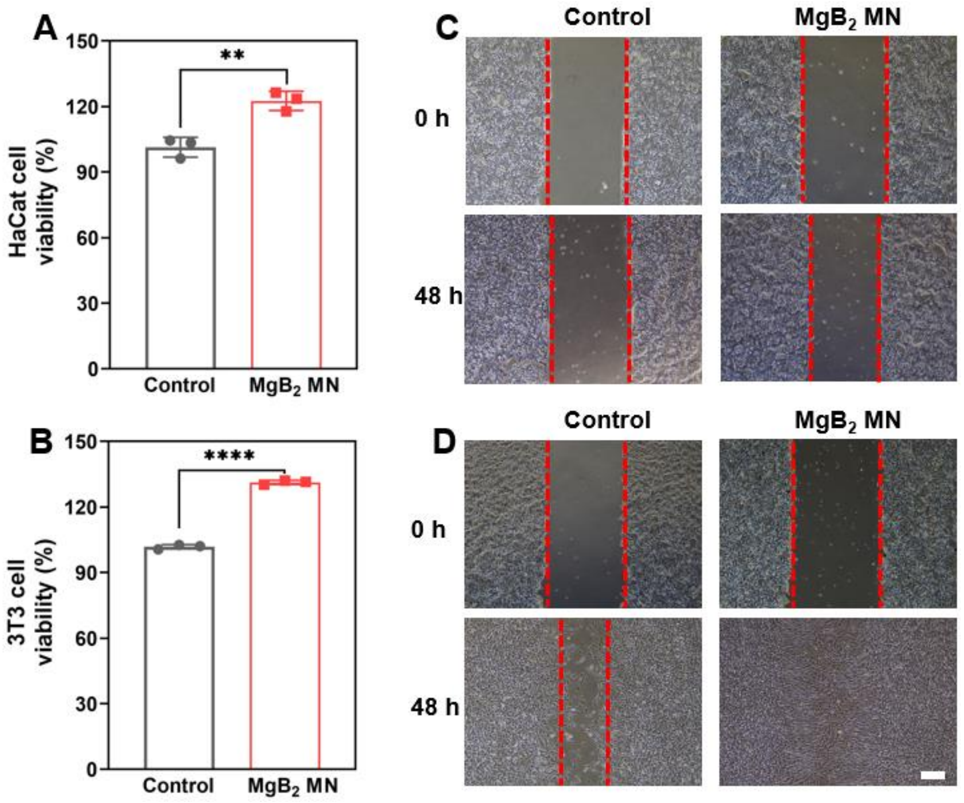


**Figure S12.** Viability of (A) keratinocytes (HaCat) and (B) fibroblasts (3T3) after incubation with empty MN (control) and $MgB_2$ MN for 48 h, respectively. (C, D) Scratch assay images of (C) HaCat cell and (D) 3T3 cell cultivated in the medium supplementary with empty MN (control) and $MgB_2$ MN, respectively. Scale bar, 200 μm. Data represents mean ± SD, ****p < 0.0001, **p < 0.01, n = 3.

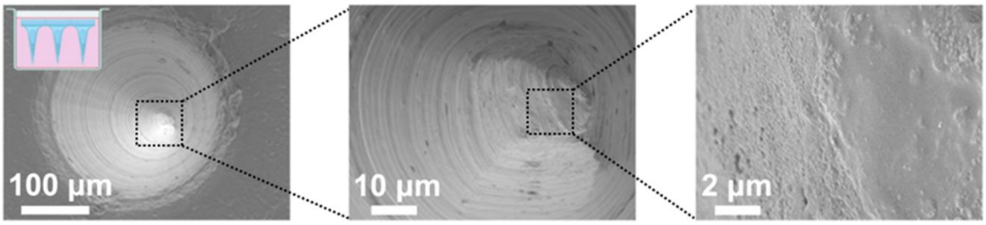


**Figure S13.** SEM image of $MgB_2$ MN after incubation in saline for 24 h.

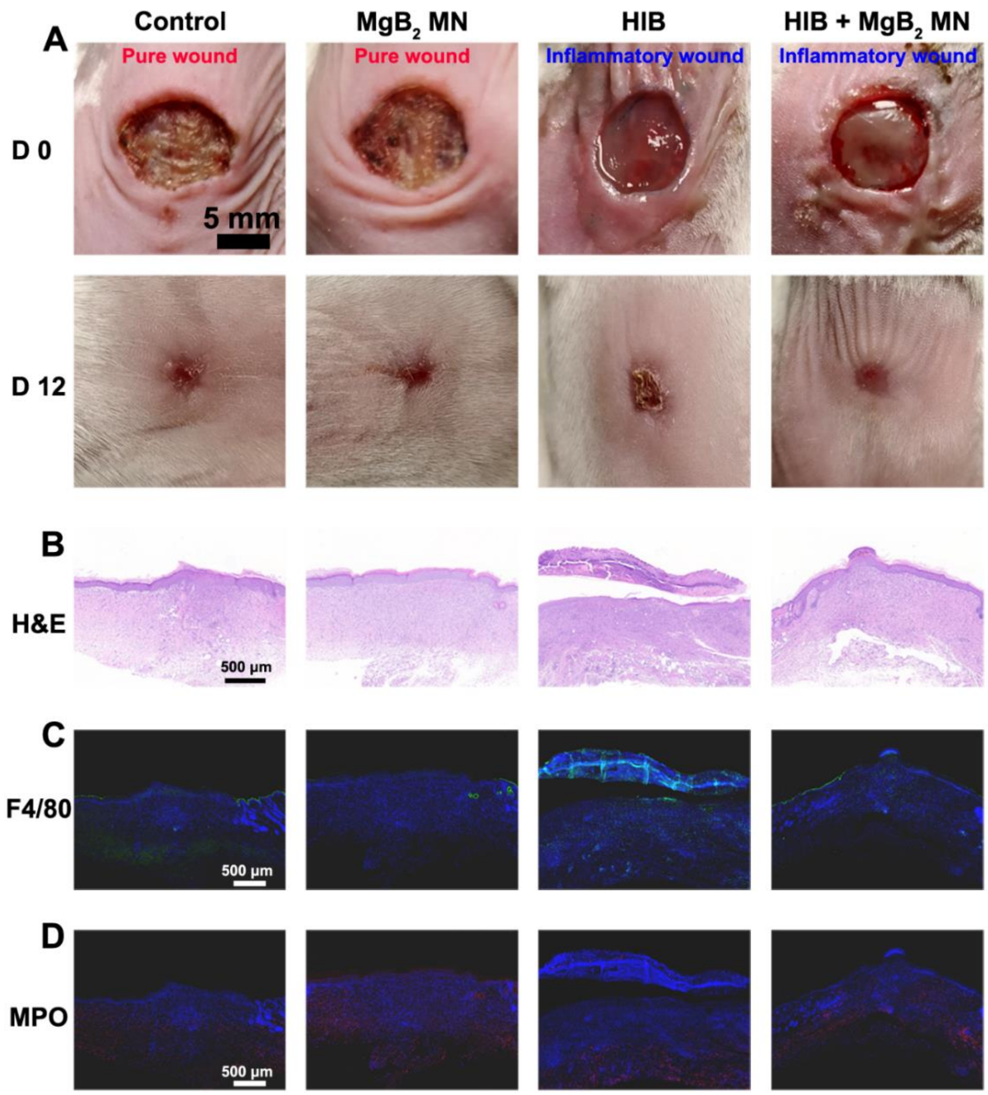


**Figure S14.** Treatment of dead bacteria-induced wound inflammation. (A) Photographs of dead bacteria-induced wound inflammation treated with or without $MgB_2$ MN. (B) H&E staining of HIB (dead MRSA)-induced wounds. Immunofluorescence staining with the (C) macrophage cell marker F4/80 and (D) neutrophil cell marker myeloperoxidase (MPO) of HIB-induced wounds.
